# The role of gut microbiota on cognitive development in rodents: a meta-analysis

**DOI:** 10.1186/s12576-023-00869-1

**Published:** 2023-05-16

**Authors:** Siti Sarahdeaz Fazzaura Putri, Irfannuddin Irfannuddin, Krisna Murti, Yudianita Kesuma, Hardi Darmawan, Noriyuki Koibuchi

**Affiliations:** 1 Faculty of Medicine, Universitas Sriwijaya, Palembang, 30126 Indonesia; 2 Department of Integrative Physiology, Gunma University Graduate School of Medicine, Maebashi, Gunma 371-8511 Japan

**Keywords:** Gut microbiota, Cognitive development, Probiotic, Prebiotic, Meta-analysis

## Abstract

Cognitive function includes learning, remembering and using acquired information. Emerging studies indicate the correlation between microbiota and cognitive function. Higher abundance of a specific gut microbiota, such as Bacteroidetes may improve cognitive abilities. However, another study reported different result. These results suggest that further systematic analysis is required to determine the effect of the gut microbiota abundance on cognitive development. The aim of this study is to summarize the abundance of the specific gut microbiota and cognitive development using meta-analysis. PubMed, ScienceDirect, and Clinical-Key were used as data bases to perform the literature search. Phylum Bacteroidetes, and family Lactobacillaceae were more abundant in cognitive-behavioral enhancement (CBE), whereas Firmicutes, Proteobacteria, Actinobacteria, and family Ruminococcaceae were less abundant in CBE. Differences in gut microbiota abundance are influenced by differences in stage of cognitive dysfunction, intervention, and strain of gut microbiota.

**Supplementary Information:**

The online version contains supplementary material available at 10.1186/s12576-023-00869-1.

## Background

Cognitive disorder is a condition of diminished ability of learning, remembering and using acquired information [1]. Cognitive disorders in childhood affect greatly their behavior and sometimes require special educational resources [2]. In the elderly, on the other hand, cognitive disorders might increase the risk of dementia [3]. Impaired cognitive development causes diverse behavioral and neuro-psychological characteristics, but the mechanisms causing such characteristics are not still well known. Previous studies proposed genetic and epigenetic influences, such as chromosomal abnormalities, brain injuries and inflammation, and environmental chemical exposure [4, 5]. Moreover, recent studies have proposed that gastrointestinal impairment may be also associated with impaired cognitive development [6]. The gastrointestinal impairment can be caused by metabolic diseases, enteric nervous system disorders, and immune disorders [7].

The central nervous system has bidirectional communication with the gastrointestinal tract. This communication is known as the microbiota–gut–brain axis [8]. Through such axis, information from gastrointestinal tract affects brain development, including cognitive development [9]. Although the substance produced by microbiota and involved in cognitive function has not yet been clarified, one candidate may be short-chain fatty acids (SCFAs) [10]. The alteration of the gut microbiota affects SCFAs production [11]. Complex carbohydrates such as prebiotic supplementation and dietary fibers were fermented in the colon by the gut microbiota into SCFAs such as n-butyrate, acetate and propionate [12]. SCFAs enter the circulation and cross the blood brain barrier (BBB) [13]. These SCFAs may enhance the integrity of BBB [14]. Therefore, increased transport of molecules and nutrients from the circulation to the brain, can trigger its growth and development [15].

The hippocampus play an important role in controlling cognitive function [16]. The hippocampus is an area of the brain that consistently maintains its ability to generate neurons throughout life [17]. Previous studies reported the functional development of the hippocampus play an important role in the process of learning and memory [16, 17]. Cognitive impairment has been widely associated with neuronal atrophy in the hippocampus [18]. This condition is associated with dysbiosis of the gut microbiota [18, 19]. A higher abundance of specific gut microbiota, such as Bacteroidetes may improve cognitive abilities [19]. However, another study reported different results. For example, Wang et al. showed better cognitive ability in the group with a lower percentage of Bacteroidetes [20]. Additionally, the differences of gut microbiota between cognitive-behavioral enhancement (CBE) and non-CBE were also not determined.

Based on these studies, further systematic analysis is required to determine the abundance of the gut microbiota on cognitive development. For such purpose, a meta-analysis was conducted to analyze the abundance of the specific gut microbiota on cognitive function in rodents model.

## Methods

Present review is reported according to the Preferred Reporting Items for Systematic Reviews and Meta-Analyses (PRISMA) 2020 guidelines [21] (Additional file 1).

### Eligibility criteria

To be included in present meta-analysis, studies must meet the following inclusion criteria: (1) using rat/mice/mouse/*Mus musculus*/*Rattus* as the population; (2) reported outcome on cognitive behavior test; (3) presented the bacterial information including bacterial taxonomy and proportion; and (4) used pre-clinical as study design.

### Information sources

Online databases including PubMed, ScienceDirect, and ClinicalKey were used to perform the literature searched to identify eligible studies without any year restrictions until June 1st, 2021. The population (P) of this meta-analysis was rat or mice performing cognitive-behavioral test, while the outcome (O) was gut microbiota abundance measurement.

### Search strategy

The following search terms for the population: (rat OR mice OR mouse OR Mus musculus OR Rattus) AND (cognitive OR neurogenesis OR neurocognitive OR memory OR recognition OR proliferation OR plasticity); these were combined with terms relevant to the outcomes: (gut microbiota OR enteral microbiome OR enteral microbiota). Only articles written in English were selected.

### Selection process

After the inclusion criteria were specified, two independent reviewers (SS and II) initiated the screening process. First, the titles and abstracts were screened to identify eligible studies. Second, SS and II screened the remaining articles for full-texts detailed assessment. Third, the group with statistically increased cognitive behavior (*P* < 0.05) based on the cognitive-behavioral test was designated as the CBE group. And then, for consistency, the included studies were analyzed at the phylum and family level. Any disagreements on the eligibility of the studies were resolved with a third reviewer (NK).

### Data items and collection process

The following information were extracted eligible studies: year of publication, authors, rodent species and strain, sex, sample size, age of testing, types of cognitive-behavioral test, studies intervention and result of studies. Data extraction was independently performed by the two reviewers (SS and II). If any relevant data were presented in graph, WebPlotDigitizer was used to convert graphically represented data into numerical values [22]. The results were verified by a third reviewer (NK).

### Risk of bias assessment

The risk of bias of the included studies was evaluated using the SYstematic Review Center for Laboratory Animal Experimentation (SYRCLE)’s Risk of Bias (RoB) tool [23]. Two of us (SS and II) independently rated the studies as having “low”, “unclear”, and “high” risk of bias in six dimensions: sequence generation, baseline characteristics and allocation concealment (selection bias), random housing and blinding (performance bias), random outcome assessment and blinding (detection bias), incomplete outcome data (attrition bias), selective outcome reporting (reporting bias), and other sources of bias (other). Disagreements in scores were resolved through discussion with a third reviewer (NK).

### Statistical analysis

Effect size used in this meta-analysis was the proportion of percentage (%) of the gut microbiota abundance, if more than 5 studies were included in the analysis, the random-effects model were used. Otherwise, the fixed-effects model would be selected [24]. Forest plots were used to visualize the result of analysis. Statistical heterogeneity was assessed using the *I*^2^ index. Indeed, all statistical analyses were carried out using STATA 16.0 (USA).

## Results

**Fig. 1:**
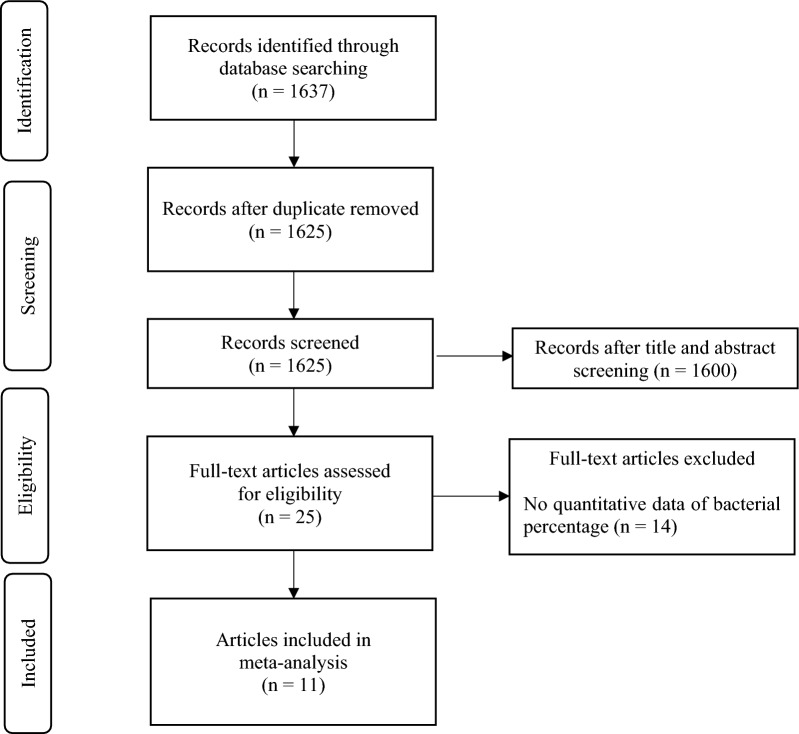
Study selection flow

At initial search, 1637 articles were identified for consideration in the present meta-analysis. After exclusion of 12 duplicate reports, 1625 abstracts were reviewed. Twenty-five articles were assessed for eligibility in this meta-analysis. Exclusion criteria of studies are identified in Fig. 1. We included 11 papers with a total of 15 intervention arms showing CBE. Although the degree of the enhancement varied among groups, we have defined as CBE when a significant enhancement was observed by the intervention. In addition, although the method of invention was different among groups, we have recruited all data showing CBE with the measurement of microbiota. The characteristics of the included studies are described in Table 1.

**Table 1:** Characteristics of included studies

References	Species tested	Age of testing	Sample size	Cognitive behavior test	CBE group	Non-CBE group	Results
Shi et al. [25]	C57Bl/6 J male mice	27w	*n* = 6 per group	The nesting behavior; temporal order memory tests	Mice fed with a diet rich in microbiota-accessible carbohydrates (MACs) based on the high fat: mixed with 316 g/kg from fat (soybean oil 56 g and lard 260 g) and LabDiet 5010 powder 634 g/kg	Mice fed with a diet with high fat (315 g/kg from fat: soybean oil 55 g and lard 260 g) and fiber-deficient (50 g/kg cellulose)	MACs improve cognitive impairments via the gut microbiota–brain axis induced by the consumption of a high fat diet
Yang et al. [26]	Sprague Dawley (SD) male rats	35w	*n* = 5 per group	Novel object recognition test	Mice with abdominal surgery and administered with Prebiotic Bimuno^®^ galactooligosaccharide (B-GOS) solution	Mice with abdominal surgery and administered with normal drinking water	Administration of B-GOS has a beneficial effect on regulating cognitive impairment through the manipulation of gut microbiota in a rat model of abdominal surgery
Shi et al. [27]	C57BL/6 J male mice	27w	*n* = 5 per group	Object location; novel object recognition; Nesting behavioral tests	Mice fed with oat β-glucan derived from OatWell™ oat bran (CreaNutrition, Switzerland) added into the high fat diet	Mice fed with a diet with high fat (55% by energy) and fiber-deficient (50 g/kg from cellulose, 5% fiber by weigh)	β-glucan improves indices of cognition and brain function through manipulation of gut microbiota
Jena et al. [19]	C57BL/6 male mice	32w	*n* = 7 CBE group; *n* = 4 for non-CBE group	Open field behavior study	Mice fed with fructose, palmitate, and cholesterol (FPC) enriched diet constituting 29% fat, 34% sucrose, and 1.25% cholesterol (Envigo, Indianapolis, IN, USA) plus inulin (6%, Montclair, CA, USA) supplementation	Mice fed with FPC enriched diet plus 42 g/L glucose and fructose (55%/45%)	Inulin supplementation prevented cognitive deficit caused by FPC intake via microbiota and metabolites alteration
Wang et al. [28]	C57BL/6 J male mice	20w	*n* = 30 per group	Nest building test; novel object recognition test; Morris-water maze test; Shuttle-box test	Mice fed a standard chow diet	Mice fed a chow diet supplemented with 1% choline	Excessive choline intake is associated with poorer brain health and cognitive function by remodeling the intestinal microbiota
Lee et al. [33]	C57BL/6 male mice	8w	*n* = 5 per group	Y-maze; novel object recognition; Barnes maze	(a) Sham-operated mice	(a) Mice with tIsc administered with ampicillin, 1–2 g/60 kg/day	Oral administration of antibiotics can deteriorate cognitive impairment with gut dysbiosis in ischemic brain
					(b) Mice with transient global forebrain ischemia (tIsc)	(b) Mice with tIsc administered with vancomycin, 0.5–2 g/60 kg/day	
Hsieh et al. [29]	Sprague Dawley (SD) male rats	16w	*n* = 5 for CBE group; *n* = 4 for non-CBE group	Morris-water maze test	(a) Offspring of mothers receiving the control diet	Offspring from mothers continuously receiving the low-iron diet	Maternal iron deficiency leads to an offspring spatial memory deficit and is associated with alternations in gastrointestinal microbiota and metabolite
					(b) Offspring of mothers receiving the low-iron diet but the control diet through the pregnancy		
					© Offspring of mothers receiving the low-iron diet during pregnancy but the control diet during lactation		
Yang et al. [30]	C57BL/6 J male mice	11w–12w	*n* = 5 per group	Temporal order memory: novel object recognition; Y-maze tests	Mice fed with high fat diet supplemented with curdlan from Alcaligenes faecailis (500 mg/kg food, Sigma-Aldrich, St. Louis, MO, United States)	Mice receiving the high fat diet (30% fat by weight)	Curdlan mitigated synaptic impairments induced by a high fat diet. Thus, curdlan, as a food additive and prebiotic, can prevent cognitive deficits induced by high fat diet via the colon–brain axis
Liu et al. [31]	C57BL/6 J male mice	14w	*n* = 15 per group	Novel object recognition test; Morris-water maze test;	Cerebral ischemia–reperfusion injury model mice were treated with the intragastric administration of 100 mg/kg baicalin	Cerebral ischemia–reperfusion injury model mice administered with physiological saline (0.1 ml/100 g)	Baicalin showed neuroprotective effects in cerebral ischemia–reperfusion injury model thorough modulation of the gut microbiota
Wang et al. [20]	C57BL/6 J male mice	15w	*n* = 15 per group	Morris-water maze test; novel object recognition test	Mice with age-matched healthy sham-operated	Mice with global cerebral ischemia induced by bilateral common carotid arteries	The gut microbiota components from mice with cerebral ischemia–reperfusion injury can alter animal behavior and brain functional connectivity
Xin et al. [32]	ICR male mice	18w	*n* = 6 per group	T maze; novel object recognition test	(a) Mice administered with phosphate-buffered saline (PBS)	Mice were provided fluoridated drinking water (100 ppm NaF) from 28 to 98 days	Lactobacillus johnsonii BS15 against fluoride-induced memory dysfunction in mice by modulating the gut–brain axis
					(b) Mice administered with probiotic Lactobacillus johnsonii BS15 (0.2 mL/day) for 28 days prior to and throughout a 70-day exposure to sodium fluoride (NaF)		

### Study risk of bias assessment

**Fig. 2:**
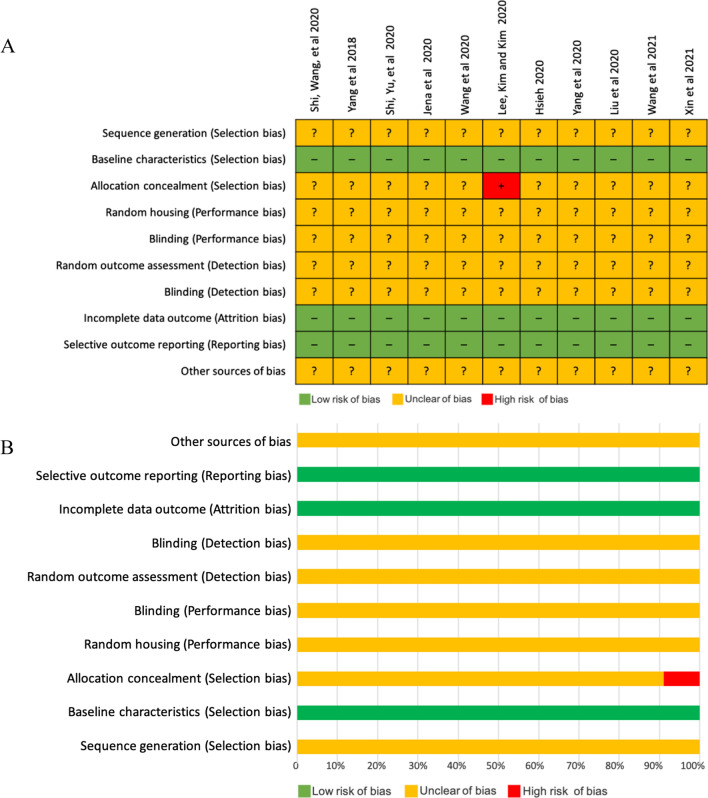
Risk of bias assessment using SYRCLE’s risk of bias tool. **A** Risk of bias summary. **B** Risk of bias graph

The risk of bias of included studies is shown in Fig. 2. All studies had detected unclear biases related to sequence generation, random housing, performance bias, and detection bias [19, 20, 25–32]. One study had high risk of allocation bias because allocation to the different groups not adequately concealed, either from allocation based on date of birth or allocation based on animal number [33].

### Abundance of phylum Bacteroidetes, Firmicutes, Proteobacteria, and Actinobacteria

**Fig. 3:**
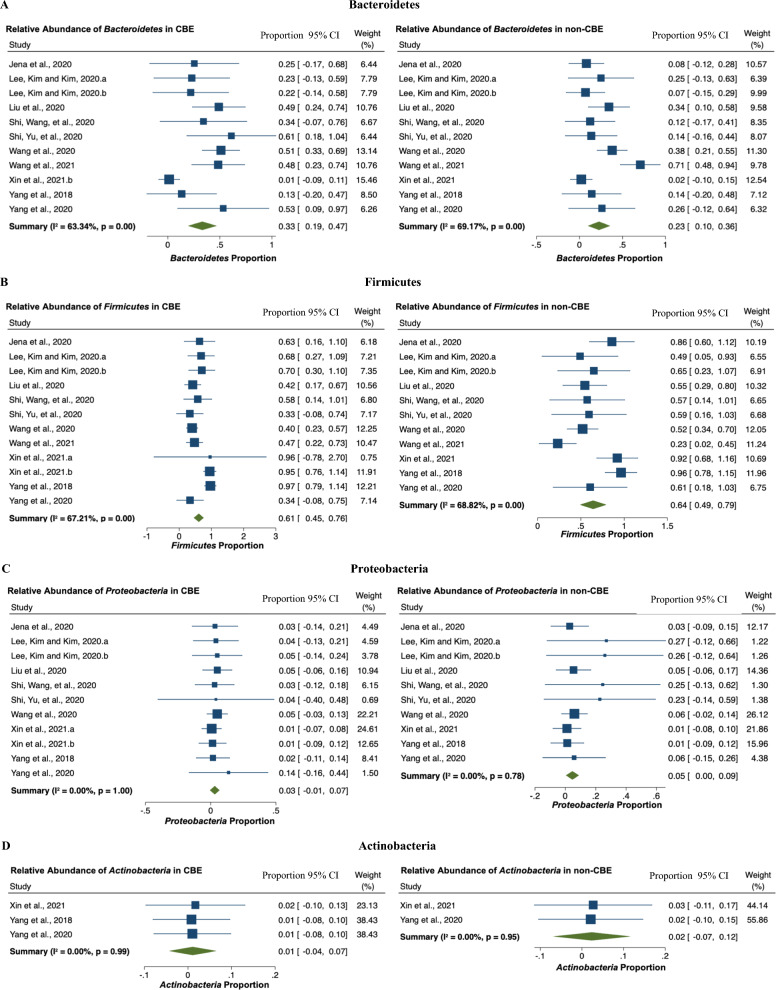
Forest plot showing the proportion of phylum Bacteroidetes, Firmicutes, Proteobacteria, and Actinobacteria in gut microbiota, in CBE and non-CBE

Bacteroidetes are highly abundant in the gastrointestinal tract [34]. Heterogeneity (*I*^2^) among studies is 63.34% in CBE and 69.17% in non-CBE (Fig. 3a). The random effect model showed the percentage of Bacteroidetes in the total gut microbiota detected was 33% in mice with CBE [95% CI 0.19–0.47], higher than non-CBE which was 23% [95% CI 0.10–0.36].

An analysis of the abundance of Firmicutes showed heterogeneity between studies (*I*^2^ = 67.21% in CBE and 68.82% in non-CBE). Random effect models showed that the percentage of Firmicutes in mice with CBE was 61% [95% CI 0.44–0.76]. However, the percentage of Firmicutes in mice with non-CBE (Fig. 3b) was 64% [95% CI 0.49–0.79].

Proteobacteria are Gram-negative bacteria that are highly abundant in the gut and most of their colonization is linked to infectious diseases [35]. Evidence of heterogeneity between studies was not found (*I*^2^ = 0%). The random effect model showed 3% of Proteobacteria were present in mice with CBE [95% CI − 0.00 to 0.07] while non-CBE accounted for 5% (Fig. 3c) of the total microbiota [95% CI 0.00–0.09].

Evidence of heterogeneity of the percentage of Actinobacteria between studies was also not found (*I*^2^ = 0%). Analysis of fixed-effects models on Actinobacteria showed 1% [95% CI − 0.04 to 0.07] in CBE. The percentage of Actinobacteria was 2% [95% CI − 0.07 to 0.12] in non-CBE (Fig. 3d).

### *Abundance of family Bacteroidaceae, Lactobacillaceae, and *Ruminococcaceae

**Fig. 4:**
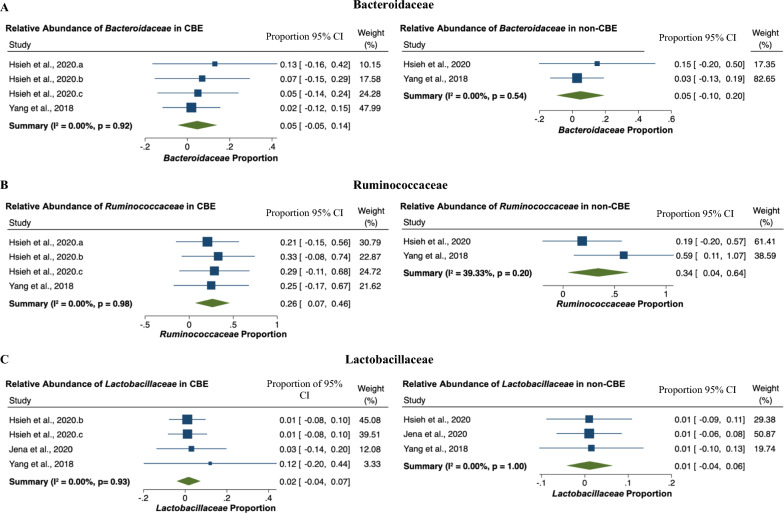
Forest plot of percentage of family Bacteroidaceae, Ruminococcaceae, and Lactobacillaceae in CBE and non-CBE

An analysis of the abundance of Bacteroidaceae and Lactobacillaceae showed no heterogeneity between studies (*I*^2^ = 0%, Fig. 4a, c). Analysis of fixed effect models showed the percentage of the Bacteroidaceae family to the total microbiota was 5% [95% CI − 0.05 to 0.14] in CBE equal to non-CBE [95% CI − 0.10 to 0.20]. These family are abundant in mammalian gut and associated in the maintenance of gut health [27, 28]. Fixed effect models showed the percentage of Lactobacillaceae in CBE was 2% [95% CI − 0.04 to 0.07] and 1% in non-CBE [95% CI − 0.04 to 0.06]. Heterogeneity between studies of the abundance of Ruminococcaceae was not found in CBE (*I*^2^ = 0%), while 39.33% in non-CBE (Fig. 4b). Ruminococcaceae percentage analysis in CBE was 26% from total gut microbiota and 34% in non-CBE (Fig. 4b).

## Discussion

The balance of the gut microbiota plays an important role in cognitive function [36]. Previous articles have reviewed the role of gut microbiota in cognitive development in humans [37, 38]. Animal research is required to further investigate the role of bacteria in modulating gut–brain interactions. However, to our knowledge, there is no article performing meta-analysis study to determine the abundance of the gut microbiota on cognitive function in rodent. In this regard, this study is the first systematic review with meta-analysis investigating the abundance of intestinal microbiota in rodents with CBE.

Phylum Bacteroidetes and family Lactobacillaceae are more abundant in CBE. Bacteroidetes and Lactobacillaceae increased in the prebiotic intervention group [19, 27, 28, 31]. Prebiotics fermentation by Bacteroidetes plays an essential role in the formation of SCFAs, which may affect the permeability of the gut and BBB [13, 39]. Furthermore, Bacteroidetes have been shown to generate acetate and propionate, which can protect neurons from oxidative damage [13]. Thus, structural and functional plasticity of the hippocampus may be in part impaired by the reduction in the percentage of Bacteroidetes [40]. In addition, Bacteroidetes also modulate the expression of BDNF, syntaxin, and drebrin in the hippocampus [41], indicating that the microbial modulation may affect behavior and cognitive performances [42]. Similarly, the Lactobacillaceae family produces butyrate, which engages in anti-inflammatory reactions and subsequently maintains the gut barrier [43, 44]. Based on a number of research, the presence of Lactobacillaceae would alter the expression of BDNF and the proBDNF proteins [45, 46]. Consequently, the Bacteroidetes and Lactobacillaceae can be regarded as a beneficial strain on brain development and plasticity.

CBE has been particularly linked to a decline in Firmicutes, Proteobacteria, Actinobacteria, and family Ruminococcaceae. A higher level of Firmicutes has consistently been observed in patients with mild cognitive impairment [47]. An increase in some bacteria belonging to phylum Firmicutes, including Ruminococcaceae, Enterococcaceae, and Streptococcaceae, have been correlated with cognitive dysfunction [47, 48]. The phylum Firmicutes has been implicated in the pathogenesis of neurodegenerative diseases [49]. Firmicutes promote an alteration in neuroactive metabolite production and modify host neurotransmitter circuitry [50]. Alteration in neurotransmitter profiles, such as glutamate, dopamine, and GABA have been implicated to the onset of neurodegenerative diseases [51]. These findings suggest that Firmicutes species may contribute to neuropathogenesis [52].

Probiotics administration can dominate certain microbiota [53]. The abundance of ileal microbes in CBE group accounts for up to 90% of the phylum Firmicutes of the total sequence due to administration of Lactobacillus johnsonii BS15 [32]. Lactobacillus johnsonii BS15 has also been identified as a possible psychobiotic, as it has been shown to avoid memory dysfunction in rats caused by psychological stress by modulating the gut environment [54]. Despite having a neuroprotective effect, Firmicutes abundance was found to be increased [32]. These findings suggests that the abundance of gut microbiota on cognitive function is also affected by the specific strains of bacteria.

Regarding other phyla, Proteobacteria and Actinobacteria were found to be less abundant in CBE. Proteobacteria at the phylum level were reported to be increase due to the administration of antibiotics followed by a decrease in the abundance of Bacteroidetes [33]. The phylum of Actinobacteria was reported to be decreased in mice supplemented with curdlan prebiotic [30]. However, Proteobacteria shown to be increase in curdlan supplemented mice [30]. The discrepancies in the findings could be caused by various animal strains, the age of the animal, or analytical methods [30, 33]. It is noticeable that neurotoxins produced by Proteobacteria associate with the production of pro-inflammatory cytokines and elevate as cognitive impairment develops [47]. Furthermore, Coriobacteriaceae bacteria from the Actinobacteria phylum were discovered to be more prevalent in mice with cognitive decline [55]. In ICR mice fed a diet low in DHA, decreased acetate and butyrate SCFAs were observed along with a rise in Actinobacteria abundance, though the specific mechanism is unclear [56]. Consequently, the Proteobacteria and Actinobacteria were regarded as unfavorable strain related to cognitive development.

Although animal models are useful to study the mechanisms of human diseases, cares should be taken on the species differences. To study the interaction between microbiome and diseases including, such concept should be also applied. Both differences and similarities exist in the composition of microbiota between humans and rodents [57]. Thus, meta-analyses in human generated similar and different findings. Patients with post-stroke cognitive impairment and depression have a higher abundance of Proteobacteria, particularly Gammaproteobacteria, Enterobacteriales, and Enterobacteriaceae [58, 59]. A meta-analysis study of the gut microbiota of Alzheimer's disease patients also revealed a considerably higher abundance of Proteobacteria [60]. These results are similar to those of animal studies showing decreased abundance in CBE. Dietary supplementation with probiotics had a highly significant effect on cognitive function in patients with cognitive impairment or Alzheimer's disease [61, 62], indicating further the importance of these bacteria. On the other hand, as stated above, while several studies showed increased abundance of Firmicutes in patients harboring cognitive impairment [47, 48], the abundance decreased in Alzheimer’s disease patients [63], indicating that the influence of Firmicutes on pathogenesis in the brain may not be consistent between species. As shown in the present study, despite a substantial number of research supporting the association between gut microbiota and cognition in rodents, it may not be adequate to extrapolate the result of rodents into humans without further studies. Unfortunately, there may be presently inadequate evidence from human studies to encourage the supplementation of specific bacteria.

Despite these remarkable findings, our study had limitations. First, there were significant statistical differences between the included studies, which could be assigned to differences in age of testing, intervention, and strain of microbiota. Nevertheless, we used the fixed-model to estimate the effect sizes in order to minimize the implications of the minimal number of studies on our results. Second, in a number of studies, we extracted the required data from bar and circle graphs, which may have resulted in another sort of bias. However, this procedure was performed by WebPlotDigitizer to convert graphically represented data into numerical values. Since we applied this methodology consistently throughout the studies, the direction of the statistical significance in the between-group comparisons would not be profoundly affected. Third, the present findings should be interpreted with caution because only a small number of studies evaluated the effects on numerous occasions. Future study should include more studies to provide greater proof on this topic.

## Conclusion

This study yielded four major insights into the nature of gut microbiota alterations in cognitive development. First, phylum Bacteroidetes, and family Lactobacillaceae were more abundant in CBE, whereas Firmicutes, Proteobacteria, Actinobacteria, and family Ruminococcaceae were less abundant. Second, Bacteroidetes and Lactobacillaceae increased in the prebiotic intervention group, while Firmicutes and Proteobacteria were less abundant. Third, administration of antibiotic resulted in an increase in the abundance of Proteobacteria and a decrease in the abundance of Bacteroidetes. Fourth, the abundance of Firmicutes dominates the gut microbe through administration of the probiotic Lactobacillus johnsonii BS15. Differences in gut microbiota abundance are influenced by differences in stage of cognitive dysfunction, intervention, and the strain of gut microbiota. Our study can contribute greatly in gaining our understanding on the role of specific bacteria on cognitive development in rodent models.

### Supplementary Information

**Additional file 1.** PRISMA checklist.

## Data Availability

The data that support the findings of this study are available from the corresponding author on reasonable request.

## Abbreviations

SD: Sprague Dawley
BBB: Blood brain barrier
BDNF: Brain derived neurotrophic factor
B-GOS: Bimuno^®^ galactooligosaccharide
CBE: Cognitive behavioral enhancement
F/B: Firmicutes/Bacteroidetes
FPC: Fructose, palmitate, and cholesterol
MACs: Microbiota accessible carbohydrates
NaF: Natrium (sodium) fluoride
Non-CBE: Cognitive behavioral enhancement
PBS: Phosphate-buffered saline
SCFAs: Short chain fatty acids
tIsc: Transient global forebrain ischemia
